# AI‐Assisted Basal Cell Carcinoma Analysis in Line‐Field Confocal OCT: Exploring Diagnostic Metrics

**DOI:** 10.1002/jbio.70374

**Published:** 2026-10-13

**Authors:** Otto Dideriksen, Kevin Jacobsen, Gavrielle R. Untracht, Peter Alshede Philipsen, Merete Haedersdal, Vinzent Kevin Ortner

**Affiliations:** ^1^ Department of Dermatology Copenhagen University Hospital—Bispebjerg and Frederiksberg Copenhagen Denmark; ^2^ Department of Health Technology Technical University of Denmark Kongens Lyngby Denmark; ^3^ Department of Clinical Medicine University of Copenhagen Copenhagen Denmark

**Keywords:** artificial intelligence, basal cell carcinoma, dermatologic imaging, keratinocyte carcinoma, line‐field confocal optical coherence tomography, noninvasive diagnostics, organ transplant recipients, skin cancer detection

## Abstract

Artificial Intelligence (AI)‐assisted line‐field confocal optical coherence tomography (LC‐OCT) may support the clinical diagnosis of basal cell carcinoma (BCC). The current model produces frame‐level BCC scores (0%–100%), but interpretation is challenging due to false positive frames. We present two novel scan‐level metrics: the composite score (percentage of frames scoring 100% per scan) and the stability score (longest sequence of consecutive 100%‐score‐frames per scan). LC‐OCT videos (*n* = 568) from 82 lesions from 52 patients were analyzed, including 18 BCCs and 64 non‐BCCs. Both scores were significantly higher for BCCs than for non‐BCCs (*p* < 0.0001). An exploratory threshold of 48 consecutive frames with 100% AI scores yielded 100% sensitivity (95% CI: 81.5%–100%) and 100% specificity (95% CI: 94.4%–100%). In conclusion, scan‐level composite and stability scores demonstrate consistent separation between BCC and non‐BCC lesions; however, external validation in independent cohorts is required before clinical implementation.

## Graphical abstract

Two scan‐level metrics derived from device‐embedded AI risk scores, the composite and stability scores, consistently separated basal cell carcinoma from non‐BCC lesions in line‐field confocal OCT. These exploratory metrics reduce the impact of isolated false‐positive frames and may support AI‐assisted diagnosis, pending external validation.

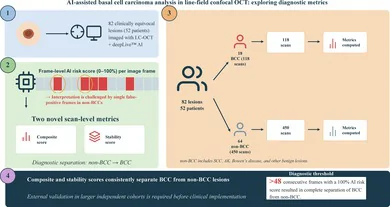

## 1 Introduction

Basal cell carcinoma (BCC) is the most common type of skin cancer, accounting for approximately 80% of cases worldwide [1, 2]. Its incidence is steadily increasing, particularly among fair‐skinned individuals exposed to high cumulative levels of ultraviolet radiation. Consequently, BCC places a major burden on patients and healthcare systems, both through time‐consuming diagnostics and long‐term management [3, 4, 5, 6]. These challenges underscore the need for a more efficient diagnostic workflow to alleviate the burden of BCC.

Today, the standard diagnosis of skin cancer relies on biopsy and subsequent histological evaluation, which, in addition to being invasive, can cause anxiety due to long waiting times. In addition to this psychological strain, biopsies alone have, in one study, been estimated to account for around 30% of the cost of any skin cancer diagnosis [7]. Consequently, non‐invasive imaging tools have been developed to improve the clinical workflow for skin cancer care. One such tool is line‐field confocal optical coherence tomography (LC‐OCT), introduced to dermatology in 2018 [8, 9]. This imaging device achieves near‐histological spatial resolution down to 500 μm in depth; thus, analysis of images comparable to histological examination (cross‐section) can be performed [9]. As proof of its use, European guidelines now include LC‐OCT alongside clinical examination for early stages of keratinocyte cancer [10].

To streamline the process from image acquisition to interpretation, LC‐OCT devices have been equipped with an artificial intelligence (AI) model for automated BCC analysis. The software automatically analyzes each image frame and assigns a corresponding AI risk score and heatmap, offering clinicians guidance during the examination. However, while LC‐OCT scanning is performed over an entire area to cover larger lesions, the AI predicts BCC risk only at the image frame level. This difference between clinical interpretation at the lesion level and model output may create challenges in arriving at an overall risk estimate for an entire scan or lesion. While a recent multicenter trial by Fischman et al. [11] showed that AI‐assisted LC‐OCT can enhance diagnostic accuracy, standalone AI performance was superior to that of less experienced LC‐OCT users, highlighting the need for simple metrics to support confident clinical decision‐making using AI‐assisted LC‐OCT for BCC diagnostics.

In response to this development, the aim of this study is twofold: first, to externally validate the device‐embedded AI model and second, to develop additional metrics that assist clinicians in decision‐making and help rule out potential false positives.

## 2 Methods

### 2.1 Study Design and Ethics

We conducted a retrospective observational study at the Department of Dermatology, Bispebjerg Hospital, using LC‐OCT imaging. We collected LC‐OCT videos under the approved study H‐21038387 and anonymized patient data, which were obtained with informed consent. Data from this study are securely stored and made available upon reasonable request.

### 2.2 Patient Cohort and Lesion Characteristics

We enrolled 52 organ transplant recipients with 82 clinically equivocal lesions. Included lesions were 18 BCCs, 8 squamous cell carcinomas (SCCs), 22 actinic keratoses (AKs), 12 cases of Bowen's disease (BD), and 22 other benign lesions such as seborrheic keratosis, solar elastosis, and papillomas. Multiple scans of each subject's lesions were obtained to cover the entire lesion area, resulting in a total of 568 LC‐OCT videos (118 of BCC, 450 of non‐BCC).

### 2.3 The LC‐OCT Imaging Device

Scans were performed using the deepLive LC‐OCT device (DAMAE Medical, Paris, France). The device emits a focused line of light onto the skin and measures the time of flight and amplitude of backscattered light. This enables vertical (cross‐sectional) and horizontal (en face) sectioning, as well as three‐dimensional (3D) imaging by acquiring stacks of horizontal sections. The system comprises a handheld probe and a workstation, allowing mode selection, depth and position adjustment, with LC‐OCT and co‐localized dermoscopic imaging.

The optical setup is based on a two‐beam interferometer with a microscope objective in each of its two arms (sample and reference arm). A broadband supercontinuum fiber laser (*SuperK EVO, NKT Photonics, Denmark*) with a central wavelength of ~800 nm illuminates the skin. A 500 μm thick glass window stabilizes the skin during scanning. The axial (vertical) and lateral (horizontal) resolutions of the LC‐OCT images are both 1.3 μm, and the field of view is 1.2 mm × 0.5 mm × 0.5 mm. Videos are captured and displayed at 8 fps. The device software facilitates patient data management, image review, and follow‐up [8, 9].

### 2.4 Acquisition Protocol

The operator performed LC‐OCT imaging by moving the probe over the lesion in a controlled lawnmower‐like scanning pattern to ensure complete coverage of both the tumor and its margins. The device was set to acquire cross‐sectional image frames, with the probe kept in continuous motion throughout the scanning procedure to maintain consistent imaging speed and minimize motion artifacts. In addition to LC‐OCT data, co‐localized dermoscopic images and video recordings of the skin surface were captured for each lesion. All imaging data were stored in individual patient files, which included a schematic representation of the lesion's anatomical location. The acquisition protocol is visualized in Figures 1 and 2.

**FIGURE 1:**
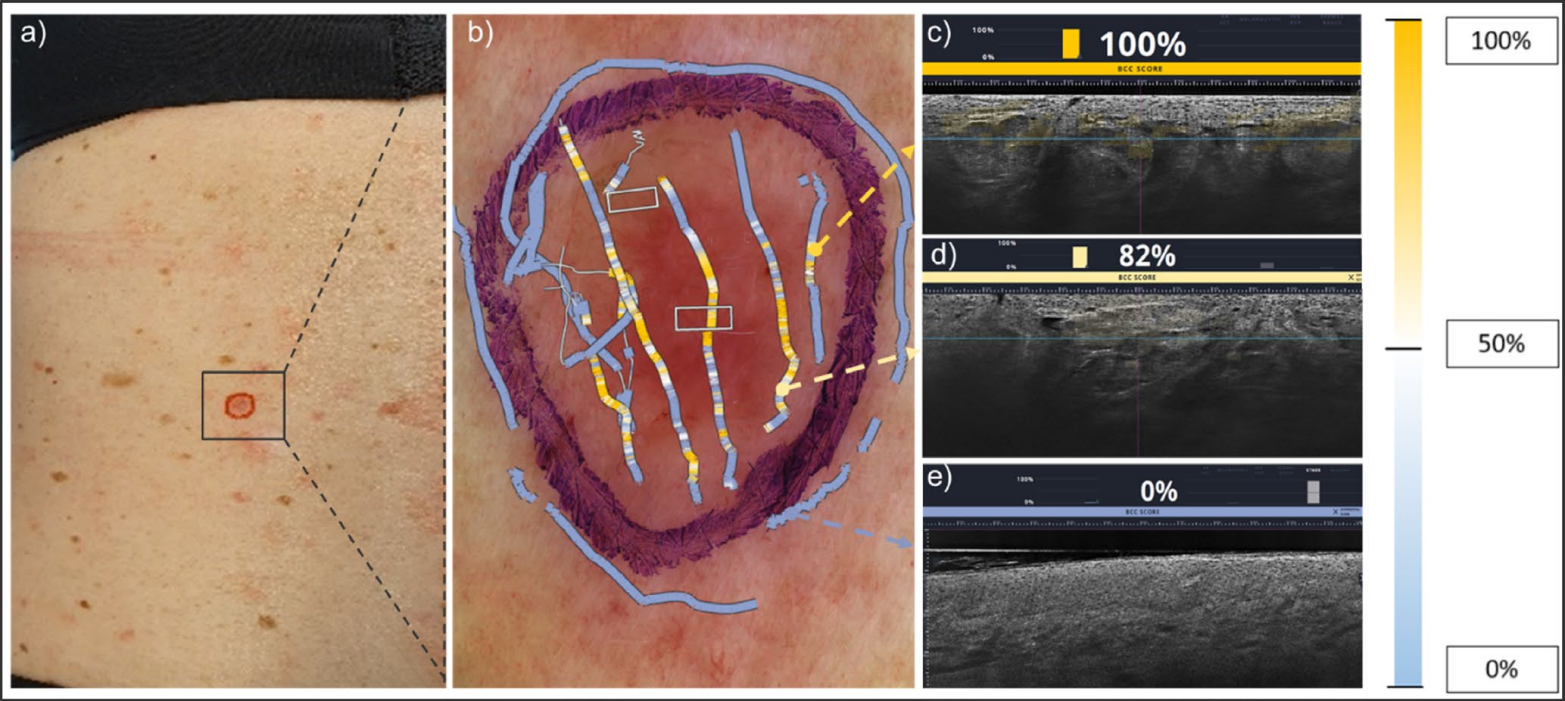
Artificial Intelligence Risk Score and Heatmap for a basal cell carcinoma. (a) Clinical image of a histologically confirmed basal cell carcinoma (BCC). (b) Dermoscopic photograph of the same lesion prior to biopsy with overlaid LC‐OCT scan trajectories. Yellow lines indicate regions where the AI predicted BCC‐like features, while blue lines indicate areas without imaging‐based BCC suspicion. AI risk scores from 0% (blue) to 100% (yellow). As shown by the blue trajectories within the tumor, some regions of a proven BCC may not be recognized by the algorithm. (c–e) Representative LC‐OCT frames with corresponding AI‐generated color‐coded risk scores. Each score reflects a single image frame, corresponding to a specific spatial point on the lesion. (c) Frame with a 100% AI risk score for BCC, (d) Frame with an 82% score. (e) Frame with a 0% score, illustrating that the algorithm did not detect any BCC‐like features at this specific point. Heatmaps (colored markings) on the images represent areas of suspicion.

**FIGURE 2:**
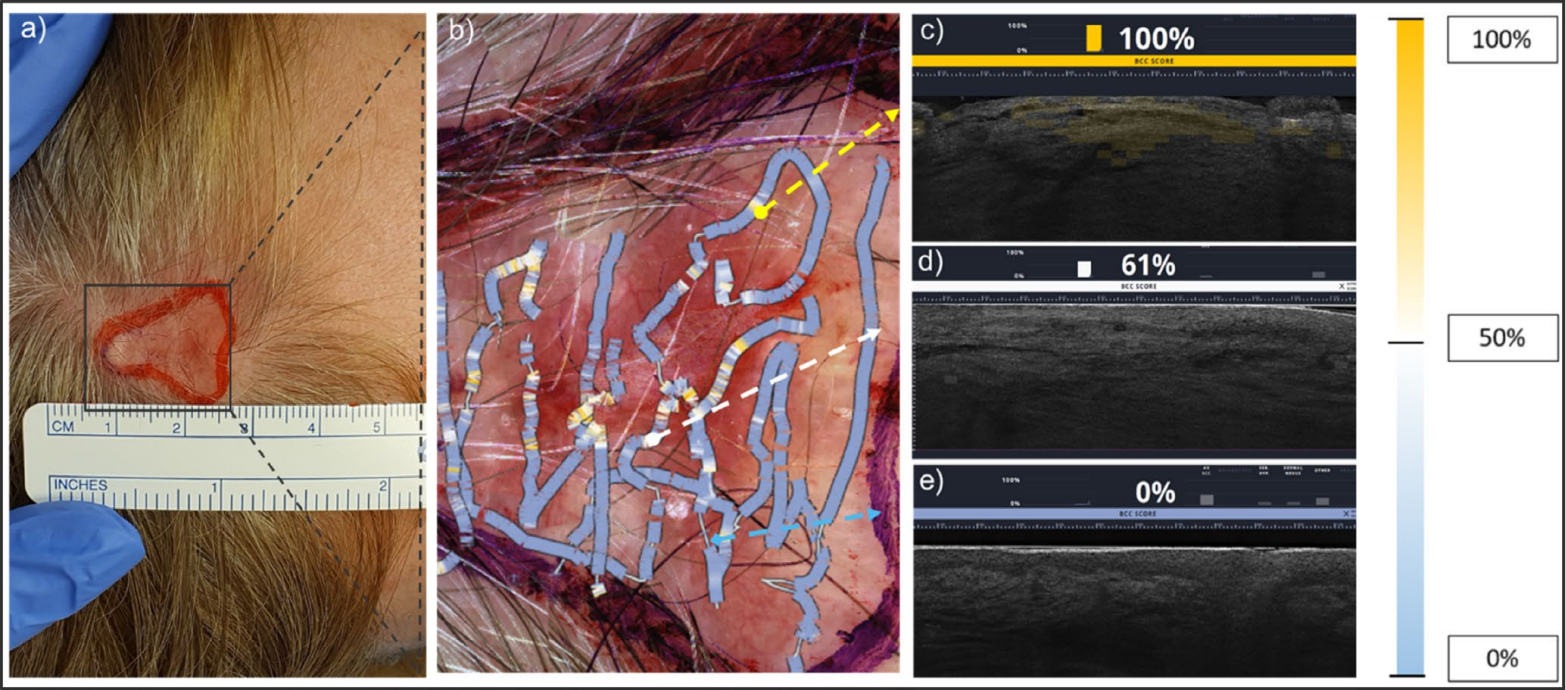
Artificial Intelligence Risk Score and Heatmap for a basal cell carcinoma mimicker. (a) Clinical image of a clinically equivocal skin lesion, histopathologically confirmed as scar tissue (cicatrice). (b) Dermoscopic photograph of the same lesion with overlaid AI‐based risk mapping derived from LC‐OCT. Blue areas indicate minimum AI confidence in BCC‐like features (0%), yellow indicates maximum confidence (100%), and intermediate colors reflect values between 1% and 99% as shown in the color bar. A few scattered segments display 100% scores despite the benign nature of the lesion, illustrating the risk of false‐positive single‐frame detections. (c–e) Representative LC‐OCT frames with corresponding AI‐generated color‐coded risk scores. Each score reflects a single image frame and corresponds to a specific spatial point on the lesion. (c) Frame with a 100% AI risk score for BCC. (d) Frame with a 61% score. (e) Frame with a 0% score, illustrating that the algorithm did not detect any BCC‐like features at this specific point.

### 2.5 Data Extraction and Processing

We extracted the data by performing screen recordings of the 568 LC‐OCT scans to capture the AI risk scores displayed during scanning. The recordings were processed in MATLAB (*MathWorks, Natick, MA, USA*), where AI risk scores were systematically extracted directly from the acquired videos and transferred to an Excel sheet for analysis. As a result, each of the 82 clinically equivocal lesions was associated with a series of scores, collectively resulting in a total of 116 396 scores.

### 2.6 AI Risk Score and Derived Metrics

The device‐embedded AI risk scores are generated by an algorithm built on a ConvNeXt‐Tiny deep learning architecture and has been trained on a dataset of more than 685 000 LC‐OCT images. The AI software is termed deepLive AI, and it has been certified as a Class IIa medical device under the *European Medical Device Regulation*. It assigns an AI‐score to each individual image frame, indicating the probability between 0% and 100% according to the likelihood that BCC is present, reflecting a point‐level (i.e., image frame) rather than lesion‐level prediction.

Two novel scan‐level metrics were derived from the extracted AI risk score, each calculated per individual LC‐OCT scan: *composite score* and *stability score* (see Table 1). Both metrics represent cumulative risks, that is, they summarize the occurrence of maximum confidence (i.e., 100% AI risk score) across a sequence of image frames across a lesion. The composite score represents the percentage of 100% outputs for each scan, calculated as the total number of 100% scores divided by the total number of image frames multiplied by 100% (range 0%–100%). Stability scores represent the maximal number of consecutive image frames with a 100% score (range 0–*N*_max_ frames), capturing the duration over which the AI output remains consistently at 100%. Composite and stability scores were calculated per individual LC‐OCT scan (scan level). For inter‐group comparisons, the median scan‐level score per lesion was used to represent each lesion (lesion level). We also calculated the proportion of non‐BCC scans receiving at least one image frame receiving a 100% AI risk score, representing false positive output.

**TABLE 1:** Overview of AI risk score and derived metrics for basal cell carcinoma.

Scores	Method	Input	Output	Description	Clinical value
AI Risk Score	Device‐embedded metric	Single image frame	0%–100% (AI risk score per frame)	Point level metric Predicts the AI‐estimated likelihood of BCC for each individual image frame, representing a single spatial point along the scan	Provides point‐level risk assessment; susceptible to isolated false‐positives and false‐negatives
Composite Score	Derived from AI Risk Score (MATLAB)	Sequence of image frames (video)	0%–100% (percentage of frames scoring 100%)	Scan‐level metric Percentage of image frames within a single scan that received an AI risk score of 100%	Reduced impact of isolated false‐positives/negatives; increased confidence across a broader lesion area
Stability Score	Derived from AI Risk Score (MATLAB)	Sequence of LC‐OCT frames (video)	0–*N*_max_ frames^a^ (longest uninterrupted sequence of 100% frames)	Scan‐level metric Longest consecutive sequence of frames with an AI risk score of 100%	Could differentiate true BCC AI signal patterns from false positives; permits calculation of diagnostic thresholds for better lesion‐level interpretability

^a^ *N*_max_ = total frames in that scan.

### 2.7 Statistical Analysis and Data Visualization

For all inter‐group comparisons, the composite and stability scores were computed per individual LC‐OCT scan (scan level) and aggregated to the lesion level using the median scan‐level score per lesion, so that each lesion contributed a single value. To compare the composite and stability scores between different lesion types in terms of statistical significance, the Mann–Whitney U‐test was used, given the nonparametric nature of the data from this study. With this test, the numeric output from the BCC data was compared one by one with data from each category of the other lesion types. A *p*‐value of < 0.05 was considered significant. In terms of descriptive statistics, we made histograms displaying the distribution of high and low confidence score outputs divided into intervals. We subsequently translated the stability score into a clinical threshold corresponding to the number of consecutive 100% score image frames needed to be certain of the BCC diagnosis. Confidence intervals for sensitivity and specificity of this threshold were calculated using the Clopper‐Pearson exact binomial method. To assess the robustness of the proposed metrics, a sensitivity analysis was performed by recalculating composite scores at AI risk score thresholds of ≥ 80%, ≥ 90%, and ≥ 95% in addition to the primary threshold of 100%. No imputation was performed, and all graphical representations were created using *Microsoft Excel Version 2508, IBM SPSS Statistics Version 29, and GraphPad Prism Version 10.6.0*.

## 3 Results

In this exploratory study, we evaluated the distribution of point‐level AI risk scores and the behavior of scan‐level composite and stability metrics, aggregated to lesion level, across 82 clinically equivocal lesions. Distinct and consistent patterns were observed between BCC and non‐BCC lesions.

### 3.1 Device‐Embedded AI Risk Scores

The AI model predicted high point‐level risk scores of up to 100% for individual image frames in both BCC (true positives) and non‐BCC lesions (false positives). These frame‐level scores were displayed as heatmaps, providing a visual representation of AI confidence across the scanned area (Figures 1 and 2). Across all BCC scans, 56% of image frames scored in the 80%–100% interval, whereas SCCs, AKs, BD, and other benign lesions showed only 1%–2% of frames in this interval. Each of the 118 BCC scans as well as 43 of the 450 non‐BCC scans received at least 1 frame with a 100% score. This means that 9.5% of non‐BCC scans contained at least one frame with a 100% score, representing the proportion of non‐BCC scans with at least one false‐positive frame.

As shown in the histogram in Figure 3, only 33% of BCC image frames fell in the low‐risk range (0%–20%), compared with 84% of SCC, 88% of AK, 93% of BD, and 81% of other benign lesion frames (Figure 3).

**FIGURE 3:**
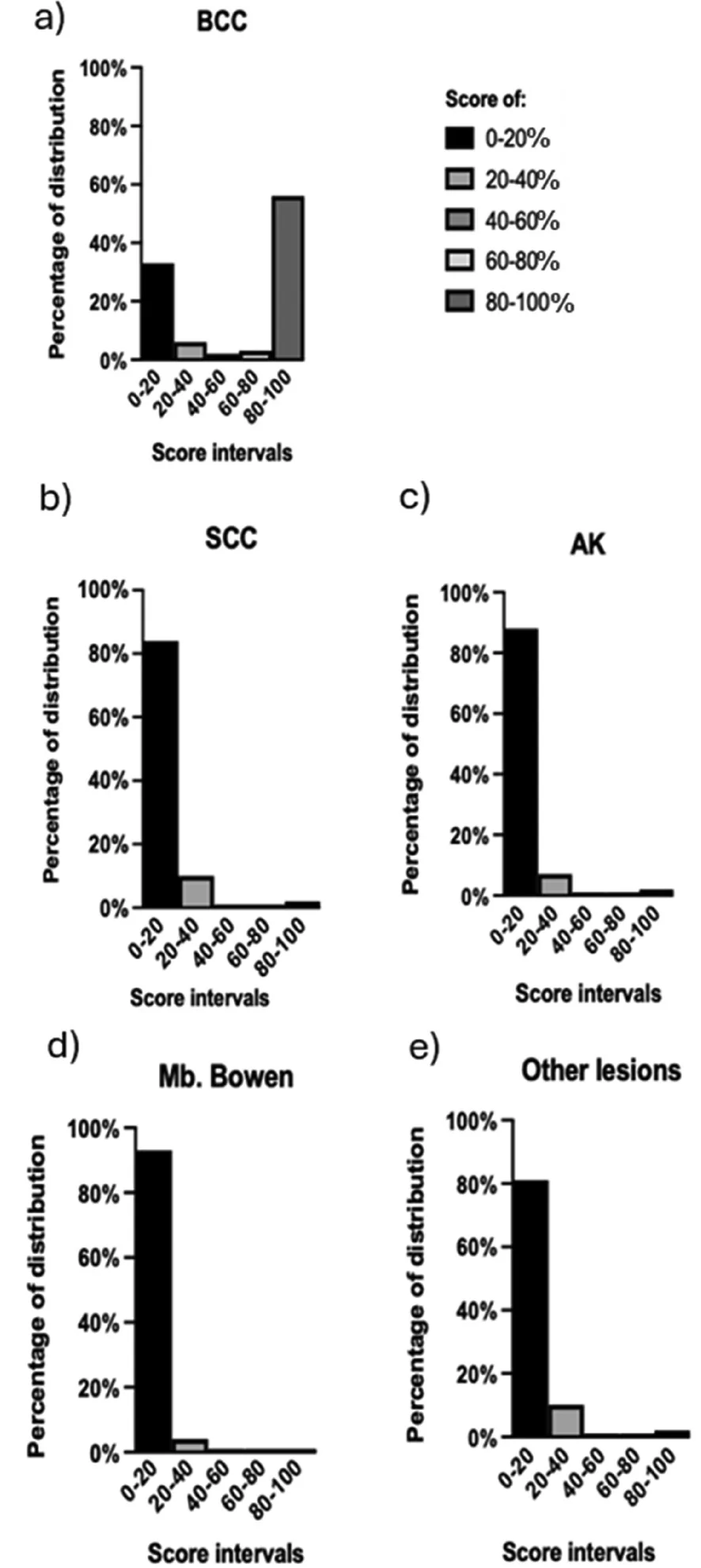
Histograms of Artificial Intelligence risk score distribution. Histograms illustrating the distribution of Artificial Intelligence (AI) risk scores reflecting the probability of basal cell carcinoma (BCC) between 0% and 100% in clinically equivocal lesions. Each panel (a–e) shows the percentage‐wise distribution of line‐field confocal optical coherence tomography (LC‐OCT) scores assigned to the multiple image frames from one lesion type. On the *x*‐axis, the scores are grouped into five intervals representing 0%–20%, 20%–40%, 40%–60%, 60%–80%, and 80%–100% AI confidence. The *y*‐axis shows the percentage of image frames falling into the respective interval for each type of lesion. (a) Histogram of BCC, (b) squamous cell carcinomas (SCC), (c) actinic keratoses (AK), (d) Bowen's Disease (BD), (e) other benign lesions. (A) BCC lesions have 56% of their total amount of image frames in the high confidence area. Panel (b–e) The control lesions had from 0% to 2% of their total amount of scores in the high confidence area.

### 3.2 Composite Score

Scan‐level composite scores were significantly higher for BCCs than for all other lesion types (Figure 4). BCC composite scores ranged from 36% to 100%, whereas AKs ranged from 0% to 18%, SCCs ranged from 0% to 5%, BD ranged from 0%, and other benign lesions ranged from 0% to 6% (*p* < 0.0001).

**FIGURE 4:**
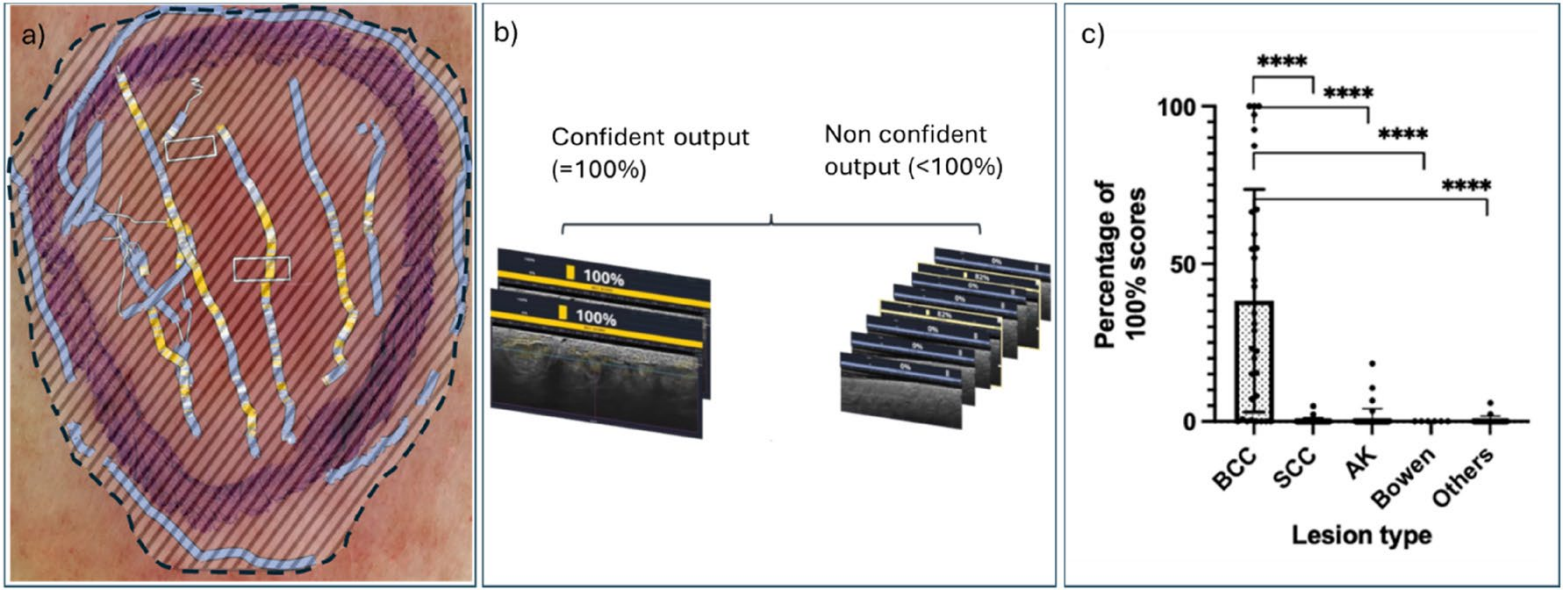
Composite score derived from AI risk scores for basal cell carcinoma. (a) Dermoscopic image of a histologically confirmed basal cell carcinoma (BCC) with overlaid LC‐OCT scan trajectories. Yellow‐highlighted segments indicate image frames where the AI model predicted 100% risk of BCC‐like features, while blue‐highlighted segments indicate frames with minimal BCC resemblance (0%). (b) Illustration of the distribution of AI risk scores from the scan shown in panel (a). The left stack represents the proportion of image frames with 100% AI risk scores, while the right stack represents frames scoring below 100%. The percentage of 100% image frames (left stack) constitutes the composite score. (c) Quantitative summary of composite scores across all lesion types. BCC lesions demonstrated the highest proportion of frames with 100% scores, whereas non‐BCC lesions showed markedly lower proportions. Each dot represents one individual LC‐OCT scan. Differences between BCC and all non‐BCC lesions were statistically significant (****p < 0.0001). Overall, BCC lesions had a median composite score of 51%, while SCC, AK, BD, and other benign lesions all had median values of 0%.

The median composite score for BCCs was 51.1% (IQR 34.2%–67.7%), while all non‐BCC lesions had median values of 0%. Thus, BCCs demonstrated a substantially higher proportion of frames with maximum AI confidence than non‐BCC lesions.

### 3.3 Threshold‐Based Sensitivity Analysis

Sensitivity analysis confirmed that the diagnostic separation between BCC and non‐BCC lesions was maintained across alternative thresholds of ≥ 80%, ≥ 90%, and ≥ 95% (Table S1). At ≥ 80%, BCC lesions showed a median composite score of 68.13% [IQR 56.05%–80.67%], while all non‐BCC groups had median scores below 1.1%. At ≥ 90%, BCC median was 66.35% [52.40%–78.76%], with all non‐BCC groups remaining below 0.6%. At ≥ 95%, BCC median was 65.17% [50.14%–76.75%], while all non‐BCC groups were below 0.4%.

### 3.4 Stability Score

BCC lesions demonstrated long, uninterrupted sequences of image frames with maximal AI risk scores at the scan level, with stability values ranging from 49 to 206 consecutive 100% frames per scan (median 152, IQR 126–173) (Figure 5). Other than BCC, AKs obtained the highest stability scores, ranging from 0 to 16 consecutive 100% frames. SCCs ranged from 0 to 13, BD showed 0 consecutive frames, and other benign lesions ranged from 0 to 9. This means that BCCs showed significantly higher values (median 152, IQR 126–173; *p* < 0.0001) compared to SCC, AK, and BD (median 0, IQR 0–0) and other benign lesions (median 0, IQR 0–1).

**FIGURE 5:**
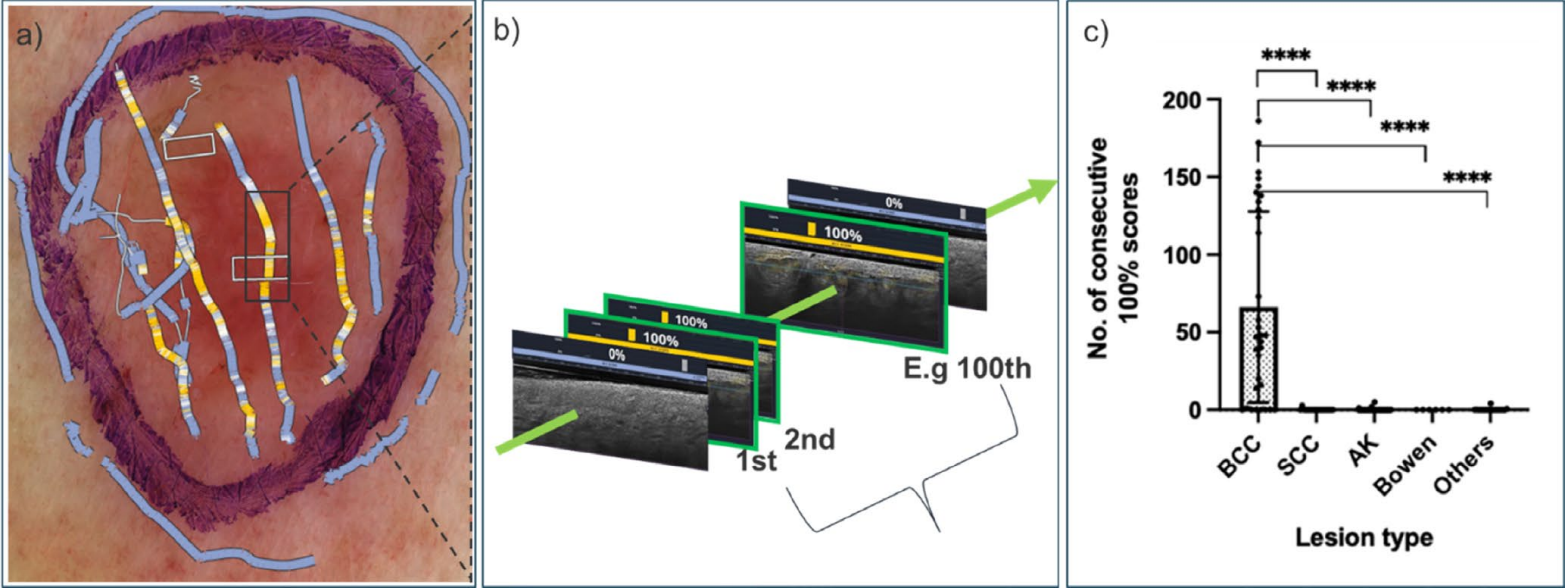
Stability score derived from AI risk scores for basal cell carcinoma. (a) Dermoscopic image of a histologically confirmed basal cell carcinoma (BCC) with overlaid LC‐OCT scan mapping. The trajectory containing the longest uninterrupted sequence of image frames with 100% AI risk scores (yellow) is highlighted. (b) Schematic close‐up of the yellow trajectory from panel (a), illustrating how individual LC‐OCT image frames are sequentially analyzed by the AI model. A continuous series of frames, for example, from the 1st to the 100th, may receive 100% AI confidence. The longest uninterrupted sequence of 100% AI risk scores within each scan constitutes the AI stability score. (c) Quantification of scan‐level stability scores across lesion types. The *y*‐axis shows the number of consecutive 100% frames per individual LC‐OCT scan, and the *x*‐axis lists histologically verified diagnoses (BCC, squamous cell carcinoma [SCC], actinic keratosis [AK], Bowen's disease [BD], and others). Each dot represents one individual LC‐OCT scan. BCCs exhibited significantly longer scan‐level sequences of 100% scores than non‐BCC lesions (****p < 0.0001). Overall, BCC lesions had a median scan‐level stability score of 152, whereas SCC, AK, BD, and other benign lesions had median values of 0.

Although a few scattered segments in non‐BCC lesions reached 100%, these occurred only as isolated events, illustrating the risk of false‐positive single‐frame detections. Only BCC lesions produced continuous, spatially coherent runs of high‐confidence scores.

### 3.5 Diagnostic Threshold for BCC

All BCC scans contained at least one image frame with a 100% AI risk score. Some non‐BCC scans displayed isolated 100% frames (SCC: 10%, AK: 7%, BD: 4%, other lesions: 9%), indicating a risk of false positives at the point level but not across entire scans.

Building on these findings, we intended to rule out these false positives by translating the LC‐OCT‐derived stability scores into a clinically relevant diagnostic threshold. The lowest number of consecutive 100% scores observed in a BCC scan was 49, while the highest number among all non‐BCC lesions was 16. Therefore, any threshold for clinicians between 17 and 48 yielded 100% sensitivity (95% CI: 81.5%–100%) and 100% specificity (95% CI: 94.4%–100%). A threshold of 48 consecutive image frames was selected as the upper boundary of this range.

## 4 Discussion

In this exploratory retrospective study of 82 clinically equivocal lesions, we validated a built‐in AI algorithm for BCC detection and introduced novel scan‐level interpretability metrics, composite and stability scores, along with an exploratory diagnostic threshold based on consecutive 100% image frames. Our results suggest that AI can identify all BCC lesions but flag a frame in almost every 10th non‐BCC scan, highlighting the potential value of scan‐level metrics for lesion‐level AI assessment.

Our metrics showed that BCC lesions had a significantly higher proportion of 100% AI risk scores (median composite score 51%, median stability score 152 image frames), and that applying a threshold of 48 consecutive image frames with 100% AI confidence, all BCC lesions were correctly identified, while none of the non‐BCC lesions reached this threshold. These exploratory findings suggest that the combined use of composite and stability scores may help distinguish BCC from non‐BCC lesions, highlighting the potential clinical utility of AI‐assisted LC‐OCT pending external validation. The choice of 100% score as the measurement for the composite score, stability score, and diagnostic threshold reflects the observed distribution of AI risk scores. As illustrated in Figure 3, scores are markedly bimodal across all lesion types, with the vast majority of frames scoring either near 0% or at 100%, and very few frames falling in the intermediate range. This pattern is consistent with our clinical experience, in which intermediate scores rarely inform diagnostic decisions in practice. We therefore based the composite score, stability score, and diagnostic threshold exclusively on frames scoring 100%.

Noninvasive diagnostic techniques and imaging modalities, including OCT, RCM, and dermoscopy, are already widely used in dermatology. Emerging research has explored the application of AI in these modalities for diagnosing BCC. The results from our cohort of 82 lesions align with the findings by Fischman et al. [11], who demonstrated that AI‐assisted LC‐OCT significantly improves diagnostic accuracy for BCC. In their multicenter study involving 43 dermatologists and 200 challenging lesions, AI assistance increased sensitivity from 84.2% to 94.7% and specificity from 89.4% to 91.9% [11].

Similarly, in our dataset, applying composite and stability metrics together with the diagnostic threshold of 48 consecutive 100% scores resulted in 100% sensitivity and 100% specificity. This indicates that cumulative lesion‐level metrics may strengthen the practical reliability of AI‐LC‐OCT in challenging clinical cases. However, these results need to be confirmed using an independent dataset to confirm their clinical utility.

### 4.1 Strengths and Limitations

In this study, we introduced novel metrics in the form of composite and stability scores to validate and assess the AI's capabilities. The composite score provides an overview of the overall score distribution for each lesion, offering robust differentiation between BCC and non‐BCC lesions, helping to identify false positives and potentially estimating tumor burden, which could be relevant for future treatment monitoring. Compared to the stability score, the composite score is less affected by operator technique, such as probe speed or movement pattern, making it more reproducible across different operators. The stability score, on the other hand, captures the consistency of AI scoring across a scan. Together, these metrics provide a nuanced assessment: the composite score aids lesion characterization and volume estimation, while the stability score highlights consistent high‐confidence regions found only in BCC.

However, there are limitations to consider. Only one highly experienced operator acquired the dataset. While this ensured consistency in data collection, the operator dependency and reproducibility of the metrics when applied by less‐experienced users require further study before clinical implementation. The composite score, while robust, masks intra‐lesion variability and does not reflect the consistency of AI predictions across consecutive image frames. The stability score is highly operator‐dependent, influenced by probe velocity, movement patterns, and scanning continuity, and fragmented lesions or interrupted scanning can reduce its reliability. Furthermore, the diagnostic threshold of 48 consecutive image frames with 100% scores clearly distinguished BCC from non‐BCC in this dataset. However, as this threshold was derived and evaluated within the same cohort of 18 BCCs, these findings should be interpreted as exploratory. The threshold of 48 was selected as the upper boundary of the 17–48 range as a conservative clinical reference point, and external validation in independent cohorts is required before it can be considered clinically applicable. The threshold should therefore be viewed as a guidance tool rather than an absolute rule, with both composite and stability scores serving as valuable assets for clinicians. Also, a methodological limitation of the present study is that multiple LC‐OCT scans were obtained from each lesion. This may inflate statistical significance due to intra‐lesion variation and is acknowledged as a limitation. Lastly, no reliable assessment of diagnostic accuracy can be performed as the scanning technique was not optimized to differentiate between true negatives (scanning beyond the clinical margin of the lesion) and false negatives within the BCC lesion.

### 4.2 Future Perspectives

AI offers potential to reduce biopsy reliance in multifocal or clinically challenging BCC cases, decreasing pathology workload, patient morbidity, and healthcare costs, where biopsies alone may account for ~30% of skin cancer diagnostic expenses [7]. By quantifying the reliability and spatial consistency of AI predictions, composite and stability scores may streamline clinical interpretation and expedite decision‐making in settings where rapid assessment is needed. Future research could evaluate the combined performance of clinicians and AI, as done in radiology [12, 13, 14], to identify areas where interpretive metrics most enhance diagnostic confidence [15, 16].

Future studies should focus on validating the AI model and the proposed metrics in larger and more diverse cohorts to ensure generalizability and minimize the risk of overfitting [17, 18, 19, 20, 21, 22, 23, 24]. Moreover, assessing inter‐operator dependency will be essential to determine the reproducibility of both the composite and stability scores under real‐world conditions. A systematic comparison between the two metrics could further clarify their respective strengths and clinical applicability. Additionally, testing the AI and metrics in more aggressive or rare BCC subtypes would provide insights into their robustness across the full clinical spectrum. Finally, evaluating whether the metrics are influenced by tumor size could help define their optimal diagnostic range and practical use in clinical decision‐making.

## 5 Conclusion

The device‐embedded AI risk scores provide point‐level, quantitative assessments that can assist in interpreting LC‐OCT scans of clinically equivocal lesions. By extending these point‐level outputs into scan‐level composite and stability scores, we observed that the distribution and continuity of AI risk scores across a scan may help distinguish BCC from non‐BCC lesions. These cumulative metrics may enhance diagnostic confidence and support clinicians in the use of AI for managing equivocal lesions. Since these results are exploratory, future work should validate these scan‐level metrics in larger, independent cohorts and further investigate score patterns to optimize clinical decision‐making.

## Author Contributions

**Otto Dideriksen:** data curation: collected the dataset; writing: original draft; visualization: contributed to visualizations; writing: review and editing. **Kevin Jacobsen:** resources: provided research materials; visualization: contributed to visualizations; validation: contributed to validation; writing: review and editing. **Gavrielle R. Untracht:** formal analysis: ran the metric for data collection; validation: contributed to validation; writing: review and editing. **Peter Alshede Philipsen:** formal analysis: helped with statistics; validation: contributed to validation; writing: review and editing. **Merete Haedersdal:** supervision: supervised the research; funding acquisition: secured funding; resources: provided resources; visualization: contributed to visualizations; validation: contributed to validation; writing: review and editing. **Vinzent Kevin Ortner:** methodology: developed methodology; supervision: supervised the research; funding acquisition: secured funding; visualization: contributed to visualizations; validation: contributed to validation; writing: review and editing.

## Funding

The study was performed as a part of the Danish Research Center for Skin Cancer (www.researchcenterforskincancer.org) and the Skin Cancer Innovation Clinical Academic Group (SCIN CAG) Greater Copenhagen Health Science Partners. V.K.O. received funding from the Capital Region of Denmark (Region Hovedstaden).

## Conflicts of Interest

The authors declare no conflicts of interest.

## Supporting information


**Table S1:** Median [IQR] of scan‐level composite scores per diagnosis group across the tested thresholds (lesion level, *n* = 82 lesions).

## Data Availability

The data that support the findings of this study are available on request from the corresponding author. The data are not publicly available due to privacy or ethical restrictions.
