# Supplementary material for: AI‐Assisted Basal Cell Carcinoma Analysis in Line‐Field Confocal OCT: Exploring Diagnostic Metrics
Source: J Biophotonics. 2026 Oct 13;19(10):e70374. doi: 10.1002/jbio.70374 (PMC13661145; doi:10.1002/jbio.70374)
Supplement: Supplementary file 1 [file JBIO-19-e70374-s001.docx]

AI-assisted basal cell carcinoma analysis in line-field confocal OCT: exploring diagnostic metrics

Otto Dideriksen^1^, Kevin Jacobsen^1^, Gavrielle R Untracht^1,2^, Peter Alshede Philipsen^1^, Merete Haedersdal^1,3*^ Vinzent Kevin Ortner^1*^

1 Department of Dermatology, Copenhagen University Hospital – Bispebjerg and Frederiksberg, Copenhagen, Denmark.
2 Department of Health Technology, Technical University of Denmark, 345b Ørsteds pl., Kongens Lyngby, 2800, Denmark
3 Department of Clinical Medicine, University of Copenhagen, Copenhagen, Denmark.

*joint last authorship

**Corresponding author:**

Otto Dideriksen, MD, PhD student
Department of Dermatology,
Copenhagen University Hospital - Bispebjerg, Nielsine Nielsens Vej 17, building 9, 2nd floor,
2400 Copenhagen NV,
Denmark.
E-mail: [otto.dideriksen@regionh.dk](mailto:otto.dideriksen@regionh.dk)

**Supplementary Material**

**Sensitivity Analysis**

*LC-OCT AI Scoring Metrics for BCC Detection*

This supplementary document presents a sensitivity analysis evaluating AI risk score thresholds, comparing the primary threshold of 100% with three lower thresholds (≥80%, ≥90%, and ≥95%). Values in Table S1 represent the median scan-level score per lesion (the median across all scans within each lesion), summarized as median [IQR] across lesions.

Table S1 shows the median proportion of frames scoring at the primary threshold of 100% and three lower thresholds (≥80%, ≥90%, and ≥95%) for each diagnosis group. Composite scores were calculated per individual LC-OCT scan (scan level), taken as the median per lesion, and summarized as median [IQR] across lesions.

BCC lesions consistently demonstrated markedly higher scan-level composite scores than all non-BCC groups across all three tested thresholds. At ≥80%, BCC lesions showed a median composite score of 68.13% [IQR 56.05–80.67%], while all non-BCC groups had median scores below 1.1%. At ≥90%, BCC median was 66.35% [52.40–78.76%], with all non-BCC groups remaining below 0.6%. At ≥95%, BCC median was 65.17% [50.14–76.75%], while all non-BCC groups were below 0.4%. These findings demonstrate that diagnostic separation is robust and not dependent on the specific choice of 100% as the primary threshold. However the sensitivity analysis at different thresholds does not alter the conclusion of this study.

**Table S1.** Median [IQR] of scan-level composite scores per diagnosis group across the tested thresholds (lesion level, n=82 lesions).

| **Threshold** | **BCC** | **Bowen** | **SCC** | **AK** | **Other** |
| --- | --- | --- | --- | --- | --- |
| **≥80%** | **68.13%**  [56.05–80.67%] | **1.08%**  [0.71–1.81%] | **0.11%**  [0.00–1.30%] | **0.14%**  [0.03–1.53%] | **0.00%**  [0.00–0.62%] |
| **≥90%** | **66.35%**  [52.40–78.76%] | **0.56%**  [0.40–0.97%] | **0.03%**  [0.00–0.96%] | **0.06%**  [0.00–1.12%] | **0.00%**  [0.00–0.62%] |
| **≥95%** | **65.17%**  [50.14–76.75%] | **0.32%**  [0.18–0.55%] | **0.00%**  [0.00–0.63%] | **0.02%**  [0.00–0.62%] | **0.00%**  [0.00–0.46%] |
| **=100%** | **51.10%**  [34.20–67.70%] | **0.00%**  [0.00–0.00%] | **0.00%**  [0.00–0.00%] | **0.00%**  [0.00–0.00%] | **0.00%**  [0.00–0.10%] |

*Values shown as median [interquartile range Q1–Q3]. Scan-level composite scores calculated per individual LC-OCT scan, taken as the median per lesion, then summarized as median [IQR] across lesions. BCC = basal cell carcinoma; SCC = squamous cell carcinoma; AK = actinic keratosis.*
